# Circulating chemerin levels in metabolic-associated fatty liver disease: a systematic review and meta-analysis

**DOI:** 10.1186/s12944-022-01637-7

**Published:** 2022-03-02

**Authors:** Qian Ren, Hongya Wang, Yan Zeng, Xia Fang, Mei Wang, Dongze Li, Wei Huang, Yong Xu

**Affiliations:** 1 Department of Endocrinology and Metabolism, The Affiliated Hospital of Southwest Medical University, Luzhou, Sichuan China; 2 Sichuan Clinical Research Center for Nephropathy, Luzhou, Sichuan China; 3 Luzhou Key Laboratory of Cardiovascular and Metabolic Diseases, the Affiliated Hospital of Southwest Medical University, Luzhou, Sichuan China; 4 Metabolic Vascular Disease Key Laboratory of Sichuan Province, Luzhou, Sichuan China; 5 Experimental Medicine Center, The Affiliated Hospital of Southwest Medical University, Luzhou, China

**Keywords:** Chemerin, Adipokines, Nonalcoholic fatty liver disease, Metabolic- associated fatty liver disease, Meta-analysis

## Abstract

**Background and objectives:**

Chemerin is a brand-new adipokine that has been linked to both inflammation and metabolic dysfunction. Even though a rising number of studies have connected chemerin to metabolic-associated fatty liver disease (MAFLD), formerly referred to as non-alcoholic fatty liver disease (NAFLD), this association has been controversial.

**Methods:**

A comprehensive literature search was undertaken up to February 1, 2022, in the PubMed, Embase, Web of Science, CNKI, WANFANG, and CBM library databases. Circulating chemerin levels were obtained and summarized using the standardized mean difference (SMD) and 95% confidence interval (CI). Subgroup and meta-regression analyses were conducted to examine the possibility of heterogeneity.

**Results:**

A total of 17 studies involving 2580 participants (1584 MAFLD patients and 996 controls) evaluated circulating chemerin levels in patients with MAFLD. The present study showed that higher chemerin levels were found in patients with MAFLD (SMD: 1.32; 95% CI: 0.29, 2.35) and nonalcoholic fatty liver (NAFL) (SMD: 0.75; 95% CI: 0.01, 1.50) compared to controls. However, circulating chemerin levels did not differ significantly in the following comparisons: nonalcoholic steatohepatitis (NASH) patients and controls (SMD: 0.75; 95% CI: -0.52, 2.03); NASH patients and NAFL patients (SMD: 0.16; 95% CI: -0.39, 0.70); moderate to severe steatosis and mild steatosis (SMD: 0.55; 95% CI: -0.59, 1.69); present liver fibrosis and absent liver fibrosis (SMD: 0.66; 95% CI: -0.42, 1.74); present lobular inflammation and absent lobular inflammation (SMD: 0.45; 95% CI: -0.53, 1.42); and present portal inflammation and absent portal inflammation (SMD: 1.92; 95% CI: -0.85, 4.69).

**Conclusions:**

Chemerin levels were considerably greater in patients with MAFLD than in controls, despite the fact that they were not significantly linked to different liver tissue lesions of MAFLD. In different subtypes of MAFLD, in comparison to healthy controls, the chemerin levels of NAFL patients were higher, whereas, there was no obvious difference in chemerin levels between NASH patients and controls. It is possible that chemerin will be used as a biomarker in the future to track the development and progression of MAFLD.

**Supplementary Information:**

The online version contains supplementary material available at 10.1186/s12944-022-01637-7.

## Introduction

Nonalcoholic fatty liver disease (NAFLD), one of the most common causes of end-stage liver disease, was redefined as metabolic-associated fatty liver disease (MAFLD) in recent years [1], and 25% of the world’s population is believed to be impacted by this disease according to current estimates [2, 3]. By 2030, it is anticipated that the prevalence of MAFLD will increase from 17.6 to 22.2% in China and from 26.3 to 28.4% in the United States, while in other European countries, such as Italy, the prevalence is nearly 30% [4]. MAFLD is primarily defined by lipid accumulation while excluding some other causes of lipid accumulation, such as severe alcohol intake, drug use, and genetic illnesses. MAFLD includes a series of disease processes with fatty deposits in the liver, varying from simple steatosis [nonalcoholic fatty liver (NAFL)] to nonalcoholic steatohepatitis (NASH), which gradually causes liver fibrosis and may eventually progress to cirrhosis/liver failure or even liver cancer [5, 6]. In addition to the effects on the liver itself, there is a lot of evidence suggesting that MAFLD may be related to metabolic diseases [obesity, type 2 diabetes mellitus (T2DM), hypertension, hyperlipidemia, metabolic syndrome] and extrahepatic tumors [7–9]. These adverse effects place a significant economic/health burden on society as a whole and humanity [10]. It is crucial to identify and diagnose MAFLD patients. Nevertheless, until now, the gold standard for diagnosing MAFLD has been liver biopsy, which has drawbacks including invasiveness, poor acceptability, sampling variability, and even the possibility of life-threatening complications [11]. Therefore, noninvasive biomarkers are essential for the identification of MAFLD.

Chemerin, also called retinoic acid receptor responder 2 (*RARRES2*) or tazarotene inducible gene 2 (*TIG2*), is a novel 14 kDa chemotactic protein that was initially found in psoriasis and later defined as a novel adipokine that is widely present in the liver and adipose tissue and closely related to multiple physiological activities, such as innate immunity, inflammation, endothelial dysfunction, metabolic disorders, and angiogenesis [12–15]. Experiments have shown that exogenous chemerin can exacerbate glucose tolerance and reduce serum insulin levels [16–18]. Emerging evidence has suggested that has been linked to a number of pro-inflammatory cytokines (such as *TNF-α*, C-reactive protein, and *IL-6*) [19, 20]. More recently, it has been established that serum chemerin levels can be used as an indicator of the efficacy of anti-inflammatory therapy for inflammatory bowel disease [21]. Chronic inflammation and insulin resistance have been identified as the most pathogenesis of MAFLD [22, 23]. Given the role of chemerin in regulating insulin levels and chronic inflammation, we boldly speculate whether its level is related to MAFLD. Several studies have shown a correlation between MAFLD and chemerin [24–34], but an association has not been found in other studies between chemerin levels and MAFLD [35–38]. Moreover, changes in serum chemerin have been reported in MAFLD patients with different subtypes by previous studies [24, 33, 36, 37, 39]. Similarly, the effects of circulating chemerin levels on hepatic steatosis, fibrosis, and inflammation have been investigated in several studies [24, 36, 39]. It’s still not clear what the role of circulating chemerin is in MAFLD, though. Here, a comprehensive review and meta-analysis was conducted to explore the link between chemerin levels and MAFLD (NAFL or/and NASH) and its different liver tissue lesions, including hepatic steatosis, liver fibrosis, lobular inflammation, and portal inflammation, which may provide clues for the diagnosis and treatment of MAFLD.

## Methods

### Methods and literature search

This meta-analysis was performed following a pre-defined plan and recorded in accordance with PRISMA guidelines [40] (see Supplementary Additional File 2), and it has been added to the PROSPERO database, which is the International Register of Systemic Reviews (No. CRD42021290744).

Researchers (QR and HYW) combed PubMed, EMBASE, Web of Science, CNKI, WANFANG, and CBM library databases from their inception up to February 1, 2022. The search terms were based on subject terms and free terms related to chemerin and MAFLD, such as (‘fatty liver’ OR ‘liver, nonalcoholic fatty’ OR ‘steatohepatitis, nonalcoholic’ OR ‘steatohepatitis, nonalcoholic’ OR ‘NASH’ OR ‘nonalcoholic fatty liver disease’ OR ‘NAFLD’ OR ‘MAFLD’ OR ‘metabolic-associated fatty liver disease’ OR ‘nonalcoholic fatty liver disease’ OR ‘nonalcoholic fatty liver’ OR ‘nonalcoholic steatohepatitis’ OR ‘steatosis’) And (‘chemerin’). The specific search strategy is shown in Supplemental Table 1. In addition, a manual search using a reference list of relevant articles was added. The search was not restricted by region or language. The reviewers resolved any disagreement through discussion.

### Inclusion and exclusion criteria

A protocol to answer two PI(E)CO questions was performed:

(1) “Are circulating chemerin levels increased in patients with MAFLD (NAFL and/or NASH) compared to healthy individuals?”

(2) “Are there differences in chemerin levels between groups of specific histological lesions in patients with MAFLD?”

The questions had the following statements:

(1) Otherwise healthy patients (Patients, P); MAFLD (NAFL or/and NASH) (Exposure, E); Non-MAFLD (Comparison, C); circulating chemerin levels (Outcome, O).

(2) MAFLD patients (Patients, P); A specific histopathological lesion of the liver (Exposure, E); No corresponding specific histopathological lesion of the liver (Comparison, C); circulating chemerin levels (Outcome, O).

Cohort studies, cross-sectional studies, and case-control studies were all considered for inclusion if they met the following standards: (1) participants in the case group were diagnosed with MAFLD (NAFL or/and NASH) by various means, including ultrasound and biopsy; (2) participants in the control group were healthy; (3) circulating levels of chemerin were reported; (4) subjects aged > 18 years were involved; or (5) investigation of the relationship of circulating chemerin with different liver histological lesions such as liver steatosis, liver fibrosis, lobular inflammation and portal inflammation.

Articles were excluded if they (1) studied chronic liver disease or cirrhosis caused by other factors, such as alcohol use, viruses, or autoimmune factors; (2) studied other liver diseases that coexist with MAFLD, such as hepatocellular tumors; or (3) were reviews, editorials, letters, case reports, comments, or conference abstracts.

### Quality assessment and data extraction

The Risk of Bias of all included studies was assessed by QR and HYW independently. The risk of bias in the literature included in this study was assessed using the Risk of Bias in Non-Randomized Exposure Studies (ROBINS-E) tool [41]. In addition, the grading of recommendations, assessment, development and evaluation (GRADE) methodology was utilized to assess the quality of our studies [42].

The extracted data included information on trial characteristics (first author, publication year, country, sample size, study design, method of chemerin measurement, sample type, and diagnosis method of MAFLD), baseline information of the participants [age, sex, body mass index (BMI), aspartate aminotransferase (AST) levels, alanine aminotransferase (ALT) levels, and homoeostasis model assessment of insulin resistance (HOMA-IR)], and chemerin levels in patients with MAFLD (NAFL or/NASH) and MAFLD specific liver histological lesions (mild, moderate and severe steatosis, presence and absence of liver fibrosis, lobular inflammation, and portal inflammation). If necessary, the corresponding author was contacted by email for more information to ensure that all relevant information was included. If the corresponding author did not respond, according to Cochrane’s books and Hozo et al. [43], the means and standard deviations were obtained by converting the median and quartile forms of the chemerin levels.

### Analysis and statistics

All statistical analyses were carried out with Stata 15.0 (StataCorp LP, College Station, TX, USA). The effect size was determined by the sample size, mean chemerin levels, and standard deviation (SD), and was presented as standardized mean difference (SMD) and 95% confidence interval (CI) of the circulating chemerin levels in comparisons between groups. Heterogeneity between studies was assessed using Cochrane *Q* statistics, and heterogeneity levels were measured using *I*^*2*^ statistics. Depending on the *I*^*2*^ values, a score of 25% indicated moderate heterogeneity, whereas values of 50% and 75% indicated medium and high heterogeneity, respectively. A random-effects model was used when *I*^*2*^ > 50%, conversely, a continuously weighted fixed-effects model was used. When the heterogeneity was high, subgroup and meta-regression analysis were used to evaluate the potential regulatory effect of the continuous variables on interstudy heterogeneity. To verify the firmness of the results, a sensitivity analysis omitting a single study from the primary analysis was conducted. Publication bias was evaluated by examining the symmetry of the funnel plot, and the significance of the symmetry was examined using Egger’s linear regression test. A *P* value < 0.05 was deemed statistically significant unless otherwise indicated.

## Results

### Literature search

**Fig. 1:**
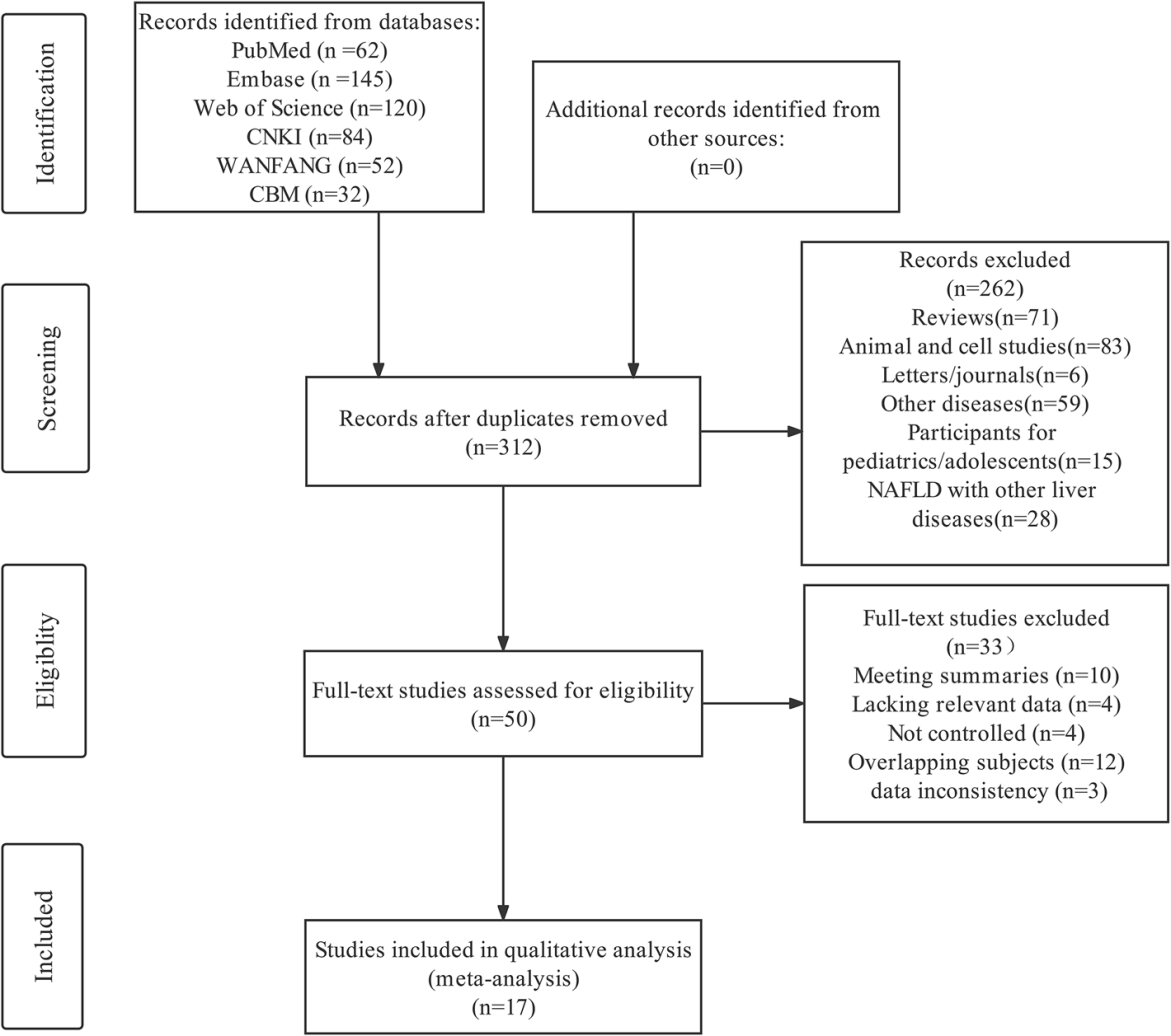
PRISMA flow chart of the literature search process

An initial search of 495 studies yielded a final total of 17 eligible reports that included 2580 participants (1584 MAFLD patients and 996 controls) that met the inclusion criteria. The results of our literature search are summarized in Fig. 1.

### Features of the included studies

Table 1 and Supplementary Table 2 contain detailed characteristics and biochemical parameters of all the study subjects. Nine studies had cross-sectional designs [26, 27, 29, 35–39, 44], and eight studies had case-control designs [24, 25, 28, 30–34]. Of all the articles, sixteen reported serum-derived chemerin levels [24–39], and only one reported plasma-derived chemerin levels [44]. These clinical tests were carried out in a variety of locations throughout the world: seven studies were conducted in Europe (Germany [36], Belgium [37], Greece [38], and France [44] conducted one each; three were conducted in Poland [24, 27, 39]); eight studies were conducted in Asia (one was conducted in Turkey [25], and seven were conducted in China [26, 28, 29, 31, 33–35]). Only two studies were conducted in Africa (Egypt [30, 32]). Eight studies used liver biopsy [24, 25, 27, 29, 36, 37, 39, 44] to identify MAFLD, six studies used ultrasound technology [26, 30, 32, 34, 35, 38], and three studies were unclear about the method of determining a specific diagnosis of NAFL [28, 31, 33]. For the circulating chemerin levels in different types and pathological features of MAFLD, fifteen studies [24–29, 31–38] focused on MAFLD patients (1484 participants) and controls (996 participants); four studies [24, 30, 36, 37] focused on NAFL patients (107 participants) and controls (85 participants); four studies [24, 30, 36, 37] focused on NASH patients (76 participants) and controls (76 participants); five studies [24, 30, 36, 37, 39] concentrated on patients with NASH (93 participants) and NAFL (146 participants); three studies [24, 36, 44] investigated patients with mild and moderate steatosis and patients with severe steatosis (71 and 69 participants, respectively); four studies [24, 36, 39, 44] assessed patients with and without liver fibrosis (101 and 71 participants, respectively); three studies [36, 39, 44] assessed patients with and without lobular inflammation (63 and 68 participants, respectively); and three studies [24, 36, 44] assessed patients with and without portal inflammation (69 and 47 participants, respectively).

**Table 1:** Details of the selected studies and baseline characteristics of the participants

Authors	Year	Country	Sample size	Study design	Method of chemerin measurement	Sample	Diagnostic methods of MAFLD
Kukla et al.	2010	Poland	61	Case-control	Enzyme-linked immunosorbent assay	Serum	Liver biopsy
Sell et al.	2010	France	44	Cross-sectional	Enzyme-linked immunosorbent assay	Plasma	Liver biopsy
Yilmaz et al.	2011	Turkey	174	Case-control	Enzyme-linked immunosorbent assay	Serum	Liver biopsy
Polyzos et al.	2014	Greece	59	Cross-sectional	Enzyme-linked immunosorbent assay	Serum	Liver biopsy
Ye et al.	2014	China	903	Cross-sectional	Enzyme-linked immunosorbent assay	Serum	Ultrasound
Zhuang et al.	2015	China	45	Cross-sectional	Enzyme-linked immunosorbent assay	Serum	Ultrasound
Zwolak et al.	2016	Poland	45	Cross-sectional	Enzyme-linked immunosorbent assay	Serum	Liver biopsy
Bekaert et al.	2016	Belgium	90	Cross-sectional	Enzyme-linked immunosorbent assay	Serum	Liver biopsy
Pohl et al.	2016	Germany	56	Cross-sectional	Enzyme-linked immunosorbent assay	Serum	Ultrasound
Lai et al.	2017	China	89	Case-control	Enzyme-linked immunosorbent assay	Serum	Liver biopsy or Ultrasound
Kajor et al.	2017	Poland	56	Cross-sectional	Enzyme-linked immunosorbent assay	Serum	Liver biopsy
Zhang et al.	2018	China	300	Cross-sectional	Enzyme-linked immunosorbent assay	Serum	Liver biopsy
Hang et al.	2019	China	310	Case-control	Enzyme-linked immunosorbent assay	Serum	Liver biopsy or Ultrasound
Sahar et al.	2019	Egypt	45	Case-control	Enzyme-linked immunosorbent assay	Serum	Ultrasound
Mohamed et al.	2021	Egypt	90	Case-control	Enzyme-linked immunosorbent assay	Serum	Ultrasound
Gao et al.	2021	China	131	Case-control	Enzyme-linked immunosorbent assay	Serum	Liver biopsy or Ultrasound
Xing et al.	2021	China	82	Case-control	Enzyme-linked immunosorbent assay	Serum	Ultrasound

*MAFLD* metabolic-associated fatty liver disease

### Outcomes

**Fig. 2:**
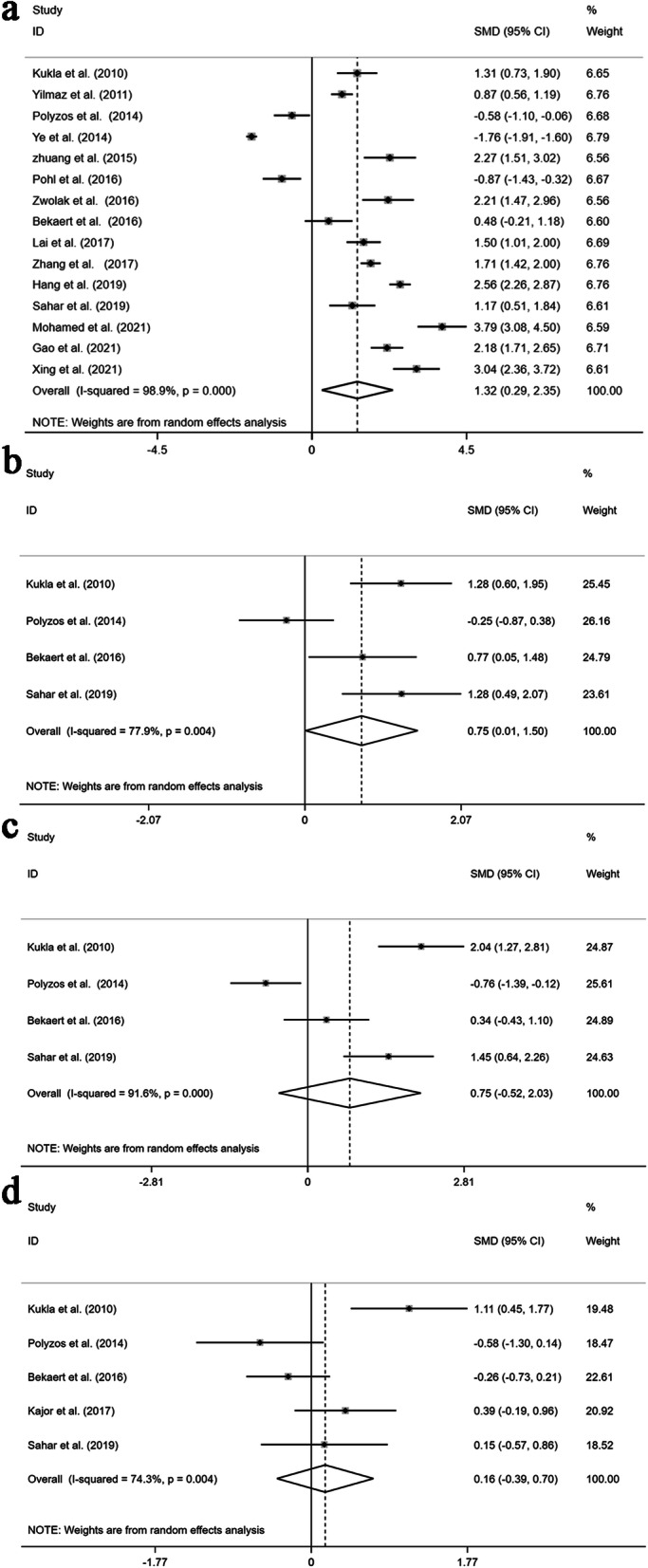
Forest plots presenting the quantitative synthesis of circulating chemerin levels, comparing the following groups in the sum of the included studies: MAFLD patients and controls (**A**); NAFL patients and controls (**B**); NASH patients and controls (**C**); and NASH patients and NAFL patients (Meta-analysis)

The random-effects model was adopted if a study had an *I*^*2*^ > 50%. The results showed that patients with MAFLD had higher chemerin levels than the control group. (SMD: 1.32; 95% CI: 0.29, 2.35, *P* = 0.012, Fig. 2A), and chemerin levels were increased in NAFL patients relative to controls (SMD: 0.75; 95% CI: 0.01, 1.50; *P* = 0.047; Fig. 2B). However, significant heterogeneity was found in these studies [Q = 1332.18, degree of freedom (df) = 14, *I*^*2*^ = 98.9%, *P* < 0.001; Q = 13.58, degree of freedom (df) = 3, *I*^*2*^ = 77.9%, *P* = 0.004]. Conversely, there were no major differences in chemerin levels in NASH patients compared to controls. (SMD: 0.75; 95% CI: -0.52, 2.03; *P* = 0.247; Fig. 2C) and NASH patients and NAFL patients (SMD: 0.16; 95% CI: -0.39, 0.70; *P* = 0.572; Fig. 2D). Nevertheless, highly significant heterogeneity was still observed among these studies [Q = 35.56, degree of freedom (df) = 3, *I*^*2*^ = 91.6%, *P* < 0.001; Q = 15.57, degree of freedom (df) = 4, *I*^*2*^ = 74.3%, *P* = 0.004].

**Fig. 3:**
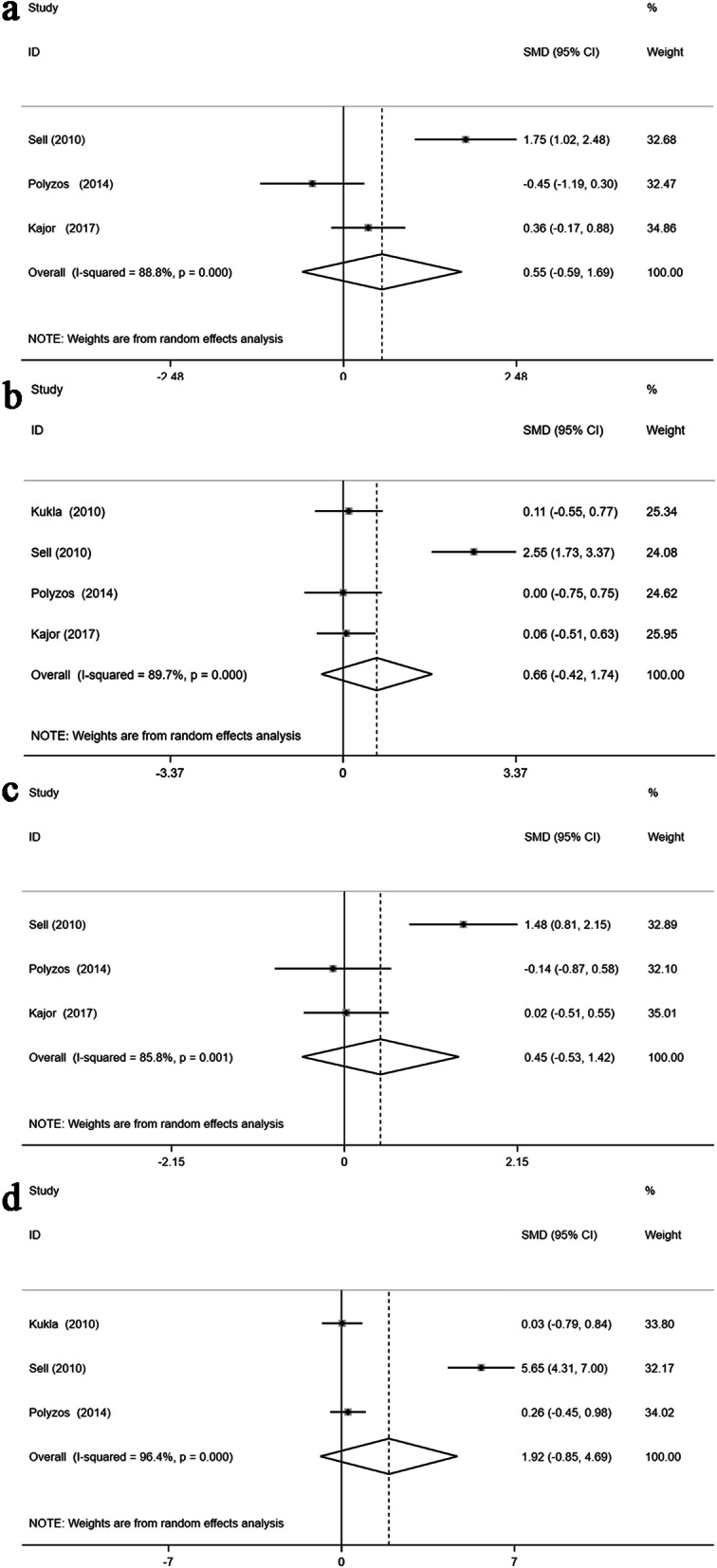
Forest plots presenting the quantitative synthesis of circulating chemerin levels, comparing the following groups in the sum of the included studies: moderate to severe steatosis and mild steatosis (**A**); present liver fibrosis and absent liver fibrosis (**B**); present lobular inflammation and absent lobular inflammation (**C**); and present portal inflammation and absent portal inflammation (**D**)

Equally interesting, there was no evident association between specific liver tissue lesions and chemerin levels in patients with MAFLD: moderate to severe steatosis and mild steatosis (SMD: 0.55; 95% CI: -0.59, 1.69; *P* = 0.344; Fig. 3A), present liver fibrosis and absent liver fibrosis (SMD: 0.66; 95% CI: -0.42, 1.74; *P* = 0.233; Fig. 3B), present lobular inflammation and absent lobular inflammation (SMD: 0.45; 95% CI: -0.53, 1.42; *P* = 0.368; Fig. 3C), and present portal inflammation and absent portal inflammation (SMD: 1.92; 95% CI: -0.85, 4.69; *P* = 0.175; Fig. 3D). Again, we observed significant heterogeneity in these studies [Q = 17.82, degree of freedom (df) = 2, *I*^*2*^ = 88.8%, *P* < 0.001; Q = 29.27, degree of freedom (df) = 3, *I*^*2*^ = 89.7%, *P* < 0.001; Q = 14.06, degree of freedom (df) = 2, *I*^*2*^ = 85.8%, *P* = 0.001; and Q = 55.40, degree of freedom (df) = 2, *I*^*2*^ = 96.4%, *P* < 0.001].

Obvious heterogeneity was found in the present study. Owing to the limited number of studies, subgroup and meta-regression analysis were performed only when patients with MAFLD were compared with controls to identify possible sources of heterogeneity. Next, based on the mean age, study design, and region, subgroup analysis was conducted. MAFLD patients over the age of 50 had significantly lower circulating chemerin levels than controls (SMD: -1.10; 95% CI: -1.93, -0.27; *P* < 0.001; Table 2); conversely, patients under the age of 50 had elevated chemerin levels when compared to controls (SMD: 1.91; 95% CI: 1.44, 2.39; *P =* 0.009; Table 2). Significant heterogeneity was found in the age subgroup analysis [Q = 25.14, degree of freedom (df) = 2, *I*^*2*^ = 92%, *P* < 0.001; Q = 126.73, degree of freedom (df) = 11, *I*^*2*^ = 91.3%, *P* < 0.001]. In the subgroup analysis of the study design, higher levels of circulating chemerin were found in MAFLD patients than in controls in the case-control studies (SMD: 1.97; 95% CI: 0.40, 3.53; *P* < 0.001; Table 2), whereas no significant differences were found in the cross-sectional studies (SMD: 0.48; 95% CI: -1.01, 1.97; *P* = 0.526; Table 2). A high degree of heterogeneity was observed in the case-control studies [Q = 109, degree of freedom (df) = 7, *I*^*2*^ = 93.6%, *P* < 0.001] and in cross-sectional studies [Q = 574.48, degree of freedom (df) = 6, *I*^*2*^ = 99%, *P* < 0.001]. There was a significant trend of high chemerin protein levels in Asian subgroups of MAFLD patients (SMD: 1.73; 95% CI: 0.35, 3.10; *P* = 0.014; Table 2), whereas chemerin levels in the others did not differ from the control group in the regional subgroup analysis (SMD: 0.50; 95% CI: -0.61, 1.60; *P* = 0.378; Table 2). A high degree of heterogeneity was still found in the regional subgroup analysis [Q = 1266.77, degree of freedom (df) = 9, *I*^*2*^ = 99.3%, *P* < 0.001; Q = 65.13, degree of freedom (df) = 4, *I*^*2*^ = 93.9%, *P* < 0.001].

**Table 2:** Subgroup analysis of circulating chemerin levels in MAFLD patients compared with controls

	All studies			
	Studies	SMD (95% CI)	*I*^*2*^*(%)*	*P* for heterogeneity
**Overall**	15	1.32 (0.29, 2.35)	98.9	< 0.0001
**Subgroup analysis**				
**Region**				
Asian	10	1.73 (0.35, 3.10)	99.3	< 0.0001
Others	5	0.50 (-0.61, 1.60)	93.9	< 0.0001
**Mean age (y)**				
<50	12	1.91 (1.44, 2.39)	91.3	< 0.0001
≥ 50	3	-1.10 (-1.93, -0.27)	92	< 0.0001
**Study design**				
Case-control	8	2.04 (1.37, 2.70)	95.5	< 0.0001
Cross-sectional	7	0.48 (-1.01, 1.97)	99	< 0.0001

*CI* confidence interval, *MAFLD* metabolic-associated fatty liver disease, *SMD* standardized mean difference

**Fig. 4:**
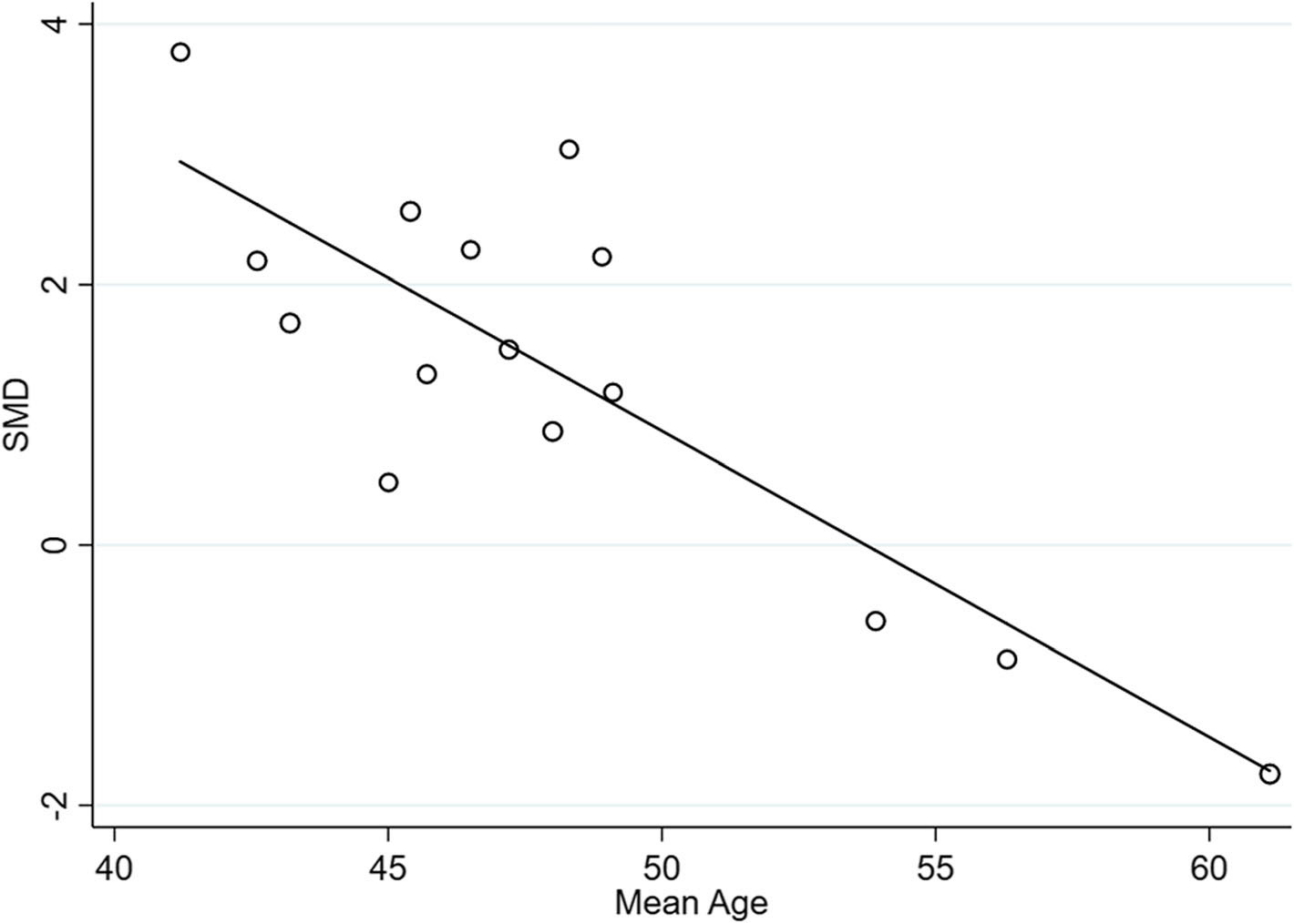
Meta-regression analysis of the effect of mean age on patients with MAFLD and healthy controls

Unfortunately, no exact source of heterogeneity was found in the subgroup analysis between MAFLD patients and controls. Next, meta-regression according to sample size, sex ratio, mean age, mean BMI, mean AST levels, mean ALT levels, and mean HOMA-IR levels in patients with MAFLD was conducted. The results suggested that the heterogeneity in the data was caused in part by the mean age of the patients with MAFLD (*R*^*2*^ = 72.08%; *β* = -0.235; *P* < 0.001; Fig. 4), and other variables did not work in the heterogeneity exploration (Supplementary Table 3).

### Study quality assessment

The risk of bias was assessed comprehensively for each of the included studies using the ROBINS-E tool (Supplementary Table 4). All qualified studies were judged to be low risk by rating the intended exposure, selection, missing data, measurement of outcomes, and reported results. Regarding confounding bias, nine studies were considered to be at serious risk [26, 27, 29, 35–39, 44], and eight studies [24, 25, 28, 30–34] were regarded as being at moderate risk. Considering exposure measurement bias, three studies were categorized as having a moderate risk of bias [28, 31, 33], and fourteen studies [24–27, 29, 30, 32, 34–39, 44] were at low risk. Overall, of all the studies, nine studies [26, 27, 29, 35–39, 44] were judged to be at serious risk, and eight studies were categorized as being at moderate risk [24, 25, 28, 30–34].

The GRADE system assessed the certainty of this study’s findings at a very low level (Supplementary Table 5). The certainty of the evidence was determined to be low due to the observational nature of the study. Among these studies, serious risk of bias as assessed by the ROBINS-E tool, serious inconsistency owing to significant heterogeneity between studies, imprecision due to the limited number of studies, and publication bias all contributed to the progressively lower certainty of evidence.

### Sensitivity and publication bias analyses

**Fig. 5:**
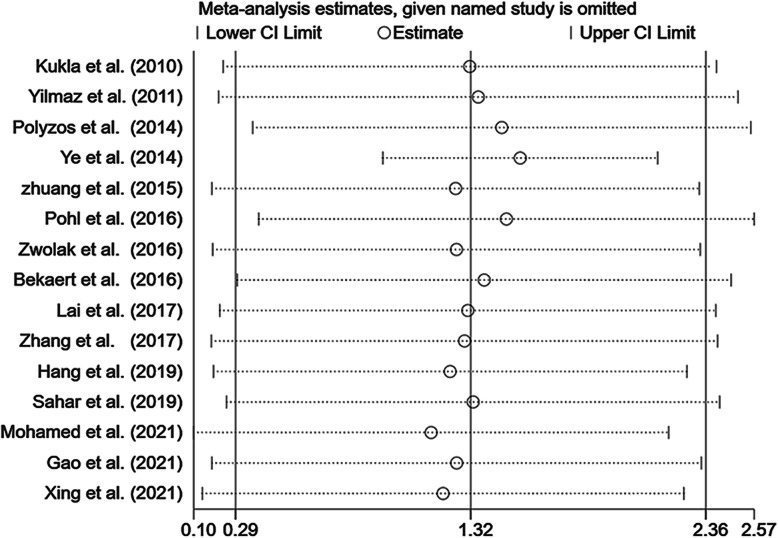
Sensitivity analysis of the summary SMD on the difference in circulating chemerin levels between MAFLD patients and controls

Due to volume limitations, sensitivity and publication bias analyses were used only for studies comparing patients with MAFLD and controls. The sensitivity analyses showed that the results of a single study had little impact on the results of this meta-analysis study (Fig. 5).

Egger’s test and funnel plots were used to determine the publication bias of the articles included in the study. The funnel plot showed significant asymmetry (Fig. S1), and Egger’s test (*P* = 0.008) indicated a potential risk of publication bias. Next, using the trim and fill method, symmetric funnel plots were created, which showed that two studies were estimated to be missing. However, the results showed a significant effect after the trim-and-fill analysis (pooled effect size: 1.001; 95% CI: 0.043, 2.049, *P* = 0.04), which confirmed the significant association of circulating chemerin with MAFLD patients in our study may not be due to publication bias.

## Discussion

There is growing concern over noninvasive diagnostic indicators for MAFLD in clinical practice. Recently, circulating chemerin levels in patients with MAFLD have gained attention. A meta-analysis of chemerin levels in patients with MAFLD has been conducted for the first time. The reasons for the differences in circulating chemerin levels between MAFLD patients and healthy subjects were further summarized.

Overall, a distinct connection was found between circulating chemerin levels and MAFLD (or NAFL), although no remarkable difference was found for NASH. Higher levels of circulating chemerin were observed in patients with MAFLD (or NAFL) than in healthy controls. Indeed, chemerin levels were found to be closely linked to liver steatosis, which is in line with previous research by Levin et al. [45]. Similarly, in the human hepatoma cell line HepG2, overexpression of chemerin causes lipid accumulation [46]. In addition, one study showed that the exogenous administration of chemerin increased lipid deposition not only in the liver, but also in the kidneys and arteries [47]. This potential association may be due to the relationship between chemerin and innate immunity, which in turn is inextricably linked to MAFLD [48, 49]. Chemerin recruits immune cells to the site of tissue damage by activating its receptor CMKLR1 [49]. In addition, there is no denying that immune cell-based cytokines in the liver are essential for hepatic steatosis pathogenesis [48]. Of greater interest was a study that suggested that chemerin may be an underlying biological marker of carotid atherosclerotic plaque damage [50], and another study pointed out that low levels of chemerin correlate with carotid plaque stability [51]. The dual effect of chemerin may explain some of the discrepancies in this phenomenon. Chemerin acts as a proinflammatory mediator; however, it can produce a potent anti-inflammatory fragment through serine (secreted by neutrophils) or cysteine protein-cleaving enzymes (released by activated macrophages) [52]. Chemerin may be primarily associated with its anti-inflammatory effects in unstable atheromatous plaques.

A high degree of heterogeneity was identified among the studies. Because of the limitations in the number of studies, only the source of heterogeneity between MAFLD and controls was explored. However, an interesting finding was that subgroup analysis revealed that chemerin levels were higher in younger patients and lower in older patients with MAFLD. Next, the results of meta-regression showed that the mean age of MAFLD patients might be a source of heterogeneity and accounted for approximately 72.08% of the sources of heterogeneity, and circulating chemerin levels were negatively correlated with the mean age of MAFLD patients. No other sources of heterogeneity were found, but this does not rule out the possibility of other causes for heterogeneity, such as the study design and different regions. As revealed in other subgroup analyses, the case-control designs showed higher levels of chemerin; in contrast, the cross-sectional studies did not find a correlation between chemerin and MAFLD patients. The same was observed for Asian patients with MAFLD who had higher levels of chemerin. However, no correlation was observed between chemerin and MAFLD patients in other continents.

What is puzzling is that an association between chemerin levels and age has also been reported in previous studies. Coimbra et al. revealed that serum chemerin levels were increased in elderly T2DM patients [53]. Researchers found that patients with chronic heart failure who had high levels of chemerin were more likely to be older [54]. This inconsistency with our results may be closely related to a nuclear receptor—farnesoid X receptor (*FXR*). *FXR* is a key regulator of lipid and glucose metabolism that is widely distributed in the liver [55], and hepatic *FXR* plays a crucial role in chemerin synthesis [56]. Patients with MAFLD had lower levels of hepatic FXR expression, according to an early investigation [57]. Subsequently, Xiong et al. noted that steatosis was exacerbated by a marked reduction in *FXR* in aging mice [58]. Therefore, we suggested that the decrease in circulating chemerin levels in elderly patients was suggested to be due to the decline of *FXR* receptor-mediated chemerin synthesis. Although liver steatosis was associated with high levels of circulating chemerin, it was possible that the extent of *FXR* downregulation in elderly patients was greater than the effect of steatosis. However, large-scale studies with prospective longitudinal designs are needed to better reveal and prove this connection.

Unfortunately, no correlation was found between chemerin levels and specific liver histological lesions. However, the use of chemerin in oncology has also been emphasized. A previous report showed that chemerin expression was reduced in hepatocellular carcinoma (HCC) tissue [59]. However, Haberl et al. noted that chemerin protein levels were increased in MAFLD-associated hepatocellular tumors [60]. This paradox may be because the majority of the former study subjects had viral hepatitis as the cause of MAFLD. Equally puzzling was the finding in animal models that serum and liver chemerin were not significantly different in NASH-associated HCC compared to NASH [61]. Therefore, further clinical studies of hepatocellular carcinoma associated with MAFLD are required to determine whether circulating chemerin could be used as a predictor of hepatocellular carcinoma in MAFLD patients.

## Study strengths and limitation

This study is the first systematic investigation of the association of chemerin with MAFLD, including different subtypes and specific hepatic histological lesions. A wide regional coverage of the population was involved, including Asia, Europe, and Africa. Chemerin was found to be a promising biomarker for the early detection of MAFLD, according to these findings. Several major limitations in the current study warrant consideration: First, the observational studies that were included only explored the association between chemerin and MAFLD and could not explain the causal relationship between them. Second, owing to the unavailability of large-scale prospective cohort studies, the relationship between chemerin levels and MAFLD should be interpreted with caution and considered hypothesis-generating. Third, despite attempts to explore the sources of heterogeneity, it was not possible to identify all possible heterogeneity because of the inherent differences in each participant, such as their complications and confounding factors (diabetes ratio, BMI, waist circumference, hip circumference, etc.), which potentially influenced their chemerin levels. Fourth, the mean values of circulating chemerin levels that were obtained from the different populations in this meta-analysis varied widely; therefore, SMD was used as a combined effect size. Furthermore, the MAFLD patients included in this study were not all diagnosed by liver biopsy; some were diagnosed by ultrasound. More studies of chemerin levels in patients with MAFLD who have been identified by biopsy are required in the future.

## Conclusions

To sum it up, MAFLD patients had higher levels of chemerin than healthy controls, suggesting that circulating chemokine levels may be a noninvasive potential biomarker in diagnostic applications for MAFLD patients. However, circulating chemerin levels were higher in younger MAFLD patients and lower in older patients. Large-scale studies are needed to assess circulating chemokine levels in MAFLD patients of different ages. In addition, chemerin levels did not differ statistically significantly between MAFLD subtypes or specific liver tissue lesions, and with the small sample size, results should be interpreted with caution because of the uncertainty. Future relevant studies with larger samples are needed to further explore the relationship.

## Supplementary information

**Additional file 1**

**Additional file 2**

## Data Availability

All data generated or analyzed during this study are included in this article. All data generated or analyzed during this study are included in this published article and its supplementary information files.

## Abbreviations

ALT: alanine aminotransferase
AST: aspartate aminotransferase
BMI: body mass index
Cl: Confidence interval
HOMA-IR: homoeostasis model assessment of insulin resistance
MAFLD: metabolic-associated fatty liver disease
NAFL: nonalcoholic fatty liver
NAFLD: nonalcoholic fatty liver disease
NASH: nonalcoholic steatohepatitis
PRISMA: Preferred Reporting Items for Systematic Reviews and Meta-analysis
RARRES2: retinoic acid receptor responder 2
SD: standard deviation
SMD: standardized mean difference
T2DM: type 2 diabetes mellitus
TIG2: tazarotene inducible gene 2
