# Supplementary material for: Circulating chemerin levels in metabolic-associated fatty liver disease: a systematic review and meta-analysis
Source: Lipids Health Dis. 2022 Mar 2;21:27. doi: 10.1186/s12944-022-01637-7 (PMC8889738; doi:10.1186/s12944-022-01637-7)
Supplement: Supplementary file 1 — Additional file 1 [file 12944_2022_1637_MOESM1_ESM.docx]

**Supplementary Table 1.** Search strategy of English database

| **Data source** | **Search terms** |
| --- | --- |
| PubMed | #1 non-alcoholic fatty liver disease [MeSH Terms]  #2 fatty liver OR liver, non-alcoholic fatty OR steatohepatitides, non-alcoholic OR steatohepatitis, nonalcoholic OR NASH OR non-alcoholic fatty liver disease OR NAFLD OR MAFLD OR metabolic-associated fatty liver disease OR nonalcoholic fatty liver disease OR nonalcoholic fatty liver OR nonalcoholic steatohepatitis OR steatosis [Title/Abstract]  #3 #1 OR #2  #4 chemerin [Title/Abstract]  #5 #3 AND #4 |
| EMBASE | (('chemerin') AND (‘fatty liver’ OR ‘liver, non-alcoholic fatty’ OR ‘steatohepatitides, non-alcoholic’ OR ‘steatohepatitis, nonalcoholic’ OR ‘NASH’ OR ‘non-alcoholic fatty liver disease’ OR ‘NAFLD’ OR ‘MAFLD’ OR ‘metabolic-associated fatty liver disease’ OR ‘nonalcoholic fatty liver disease’ OR ‘nonalcoholic fatty liver’ OR ‘nonalcoholic steatohepatitis’ OR ‘steatosis’)) |
| Web of Science | TS= (('chemerin') AND (‘fatty liver’ OR ‘liver, non-alcoholic fatty’ OR ‘steatohepatitides, non-alcoholic’ OR ‘steatohepatitis, nonalcoholic’ OR ‘NASH’ OR ‘non-alcoholic fatty liver disease’ OR ‘NAFLD’ OR ‘MAFLD’ OR ‘metabolic-associated fatty liver disease’ OR ‘nonalcoholic fatty liver disease’ OR ‘nonalcoholic fatty liver’ OR ‘nonalcoholic steatohepatitis’ OR ‘steatosis’)) |

**Supplementary Table 2.** Main demographic and biochemical characteristics of the studies included in this meta-analysis

| **Studies** | **Group** | **N**  **(Male/Female)** | **Age**  **（years）** | **BMI**  **(kg/m2)** | **AST**  **(IU/L)** | **ALT**  **(IU/L)** | **HOMA-IR** | **Chemerin** | **Additional information (Chemerin levels in different pathologic characters) (ng/mL)** | | | |
| --- | --- | --- | --- | --- | --- | --- | --- | --- | --- | --- | --- | --- |
|  |  |  |  |  |  |  |  |  | **Steatosis** | **Fibrosis** | **Lobular inflammation** | **Portal inflammation** |
| **Kajor et al.** | NAFL | 39 | NR | NR | NR | NR | NR | 863.0 ± 227.9 (ng/mL) | Mild (n = 27):  853.5 ± 234.2  Moderate to severe (n = 29):  935.1 ± 225.1 | Absent (n = 17):  884.4 ± 241.3  Present (n = 39):  897.5 ± 225.8 | Absent (n = 25):  888.1 ± 231.7  Present (n = 31)：  893.1 ± 235.3 | NR |
|  | NASH | 17 | NR | NR | NR | NR | NR | 951.6 ± 233.0 (ng/mL) |  |  |  |  |
|  | MAFLD | 56 | 39.4 ± 6.0 | 44.5 ± 8.1 | 31.1 ± 18.7 | 39.3 ± 25.4 | 8.4 ± 4.6 | 874.1 ± 234.6 (ng/mL) |  |  |  |  |
| **Kukla et al.** | Control | 20  (10/10) | 40.6 ± 5.5 | 24.4 ± 4.1 | 23.4 ± 5.9 | 21.1 ± 6.4 | 2.0 ± 0.9 | 6.1 ± 2.5 (ng/mL) | NR | Absent (n = 28):  23.2 ± 17.2  Present (n = 13):  26.3 ± 17.8 | NR | Absent (n = 7):  24.6 ± 17  Present (n = 34):  25.1 ± 19.1 |
|  | NAFL | 21 | NR | NR | NR | NR | NR | 16.3 ± 10.9 (ng/mL) |  |  |  |  |
|  | NASH | 20 | NR | NR | NR | NR | NR | 33.0 ± 18.5 (ng/mL) |  |  |  |  |
|  | MAFLD | 41  (26/15) | 45.7 ± 12.7 | 30.4 ± 3.3 | 52.8 ± 23.1 | 88.0 ± 50.8 | 3.5 ± 1.6 | 24.7 ± 17.1 (ng/mL) |  |  |  |  |
| **Pohl et al.** | Control | 32  (13/19) | 56.5 ± 16.8 | 25.6 ± 5.0 | 28.0 ± 8.0 | 20.0 ± 8.0 | NR | 117.1 ± 15.1 (ng/mL) | NR | NR | NR | NR |
|  | MAFLD | 24  (13/11) | 56.3 ± 15 | 28.5 ± 5.1 | 33.0 ± 7.0 | 26.0 ± 8.0 | NR | 106.1 ± 8.0 (ng/mL) |  |  |  |  |
| **Polyzos et al.** | Control | 28  （8/20） | 52.6 ± 8.5 | 30.9 ± 3.2 | 19.0 ± 5.3 | 20.0 ± 10.6 | 2.2 ± 1.6 | 13.3 ± 0.5 (ng/mL) | Mild (n = 29):  12.5 ± 2.7  Moderate to severe (n = 11):  11.2 ± 3.3 | Absent (n = 10):  12.1 ± 2.9  Present (n= 21):  12.1 ± 2.7 | Absent (n = 19):  12.2 ± 2.9  Present (n = 12):  11.8 ± 3.2 | Absent (n = 18):  11.8 ± 2.5  Present (n = 13):  12.5 ± 2.9 |
|  | NAFL | 15  （5/10） | 53.9 ± 10.0 | 31.9 ± 5.0 | 27.0 ± 7.8 | 42.0 ± 23.4 | 5.3 ± 9.8 | 12.9 ± 2.7 (ng/mL) |  |  |  |  |
|  | NASH | 16  （3/13） | 53.9 ± 11.6 | 34.1 ± 5.6 | 49.0 ± 36.0 | 71.0 ± 60.0 | 5.8 ± 4.4 | 11.3 ± 2.8 (ng/mL) |  |  |  |  |
|  | MAFLD | 31  (8/23) | 53.9 ± 10.9 | 33.0 ± 5.3 | 38.4 ± 26.5 | 57.0 ± 46.1 | 5.6 ± 7.5 | 12.1 ± 2.8 (ng/mL) |  |  |  |  |
| **Zhang et al.** | Control | 80  （49/31） | 42.6 ± 5.0 | 24.5 ± 2.9 | 22.3 ± 9.7 | 26.6 ± 8.1 | 4.8 ± 1.9 | 64.5 ± 9.5 (ng/mL) | NR | NR | NR | NR |
|  | MAFLD | 220  (136/84） | 43.2 ± 6.5 | 26.8 ± 2.7 | 27.5 ± 8.6 | 33.4 ± 7.7 | 8.7 ± 3.0 | 82 ± 10.5 (ng/mL) |  |  |  |  |
| **Zwolak et al.** | Control | 22  （7/15） | 47.0 ± 6.2 | 21.4 ± 2.4 | 16.5 ± 3.7 | 17.6 ± 3.6 | 1.8 ± 0.3 | 100 ± 14 (ng/mL) | NR | NR | NR | NR |
|  | MAFLD | 23  （13/10） | 48.9 ± 7.6 | 25.0 ± 2.6 | 68.0 ± 12.0 | 75.0 ± 30.0 | 3.3 ± 0.8 | 176 ± 46 (ng/mL) |  |  |  |  |
| **Bekaert et al.** | Control | 9  （2/7） | 45.0 ± 9.0 | 36.8 ± 2.1 | 19.4 ± 1.9 | 19.4 ± 5.1 | 2.1 ± 0.8 | 186.4 ± 23.7 (ng/mL) | NR | NR | NR | NR |
|  | NAFL | 56  （36/20） | 44.7 ± 11.0 | 40.9 ± 2.2 | 26.0 ± 4.7 | 23.5 ± 5.9 | 3.2 ± 1.3 | 201.2 ± 28.2 (ng/mL) |  |  |  |  |
|  | NASH | 25  （19/6） | 46.0 ± 10.0 | 41.0 ± 1.5 | 37.2 ± 7.7 | 27.9 ± 8.9 | 4.3 ± 1.6 | 194.2 ± 23.1 (ng/mL) |  |  |  |  |
|  | MAFLD | 81  （55/26） | 45.0 ± 10.0 | 41.0 ± 1.0 | 28.0 ± 3.3 | 25.0 ± 3.7 | 3.2 ± 0.7 | 199.3 ± 26.9 (ng/mL) |  |  |  |  |
| **Mohamed et al.** | Control | 30  （16/14） | 39.8 ± 7.2 | 24.3 ± 2.9 | 20.1 ± 5.5 | 19.9 ± 3.9 | 1.0 ± 0.1 | 87.6 ± 8.5 (ng/mL) | NR | NR | NR | NR |
|  | MAFLD | 60  （28/22） | 41.2 ± 7.3 | 37.6 ± 5.5 | 50.0 ± 4.4 | 49.8 ± 14.4 | 1.1 ± 0.1 | 227.4 ± 44.7 (ng/mL) |  |  |  |  |
| **Yilmaz et al.** | Control | 75  （37/38） | 48 .0 ± 7.0 | 27.4 ± 4.3 | 24.0 ± 10.0 | 21.0 ± 11.0 | 1.6 ± 0.3 | 159 ± 43 (ng/mL) | NR | NR | NR | NR |
|  | MAFLD | 99  （50/49） | 48.0 ± 8.0 | 30.6 ± 4.9 | 44.0 ± 18.0 | 68.0 ± 32.0 | 3.7 ± 0.5 | 219 ± 83 (ng/mL) |  |  |  |  |
| **Zhuang et al.** | Control | 22  (12/10) | 43.1 ± 11.7 | 21.1 ± 1.6 | 20.4 ± 3.1 | 18.6 ± 3.4 | 1.4 ± 0.4 | 113.8 ± 11.86 (ng/mL) | NR | NR | NR | NR |
|  | MAFLD | 23  (11/12) | 46.5 ± 10.2 | 23.1 ± 0.9 | 37.5 ± 6.8 | 40.3 ± 5.6 | 1.7 ± 0.4 | 134.9 ± 5.83 (ng/mL) |  |  |  |  |
| **Ye et al.** | Control | 467（206/261） | 59.3 ± 8.0 | 24.2 ± 3.1 | 22.3 ± 5.1 | 25.8 ± 5.2 | 1.6 ± 0.2 | 85.91 ± 4.8 (ng/mL) | NR | NR | NR | NR |
|  | MAFLD | 436（156/280） | 61.1 ± 8.3 | 26.6 ± 3.0 | 29.7 ± 5.1 | 35.3 ± 6.8 | 2.3 ± 0.4 | 77.84 ± 4.4 (ng/mL) |  |  |  |  |
| **Sell et al.** | Control | NR | NR | NR | NR | NR | NR | NR | Mild (n = 15):  341.0 ± 36.0  Moderate to severe (n = 29):  393 ± 26 | Absent (n = 16):  310.0 ± 33.0  Present (n = 28):  413.0 ± 44.0 | Absent (n = 24):  355.0 ± 29.0  Present (n = 20):  400.0 ± 32.0 | Absent (n = 22):  302.0 ± 26.0  Present (n = 22):  449.0 ± 26.0 |
|  | MAFLD | 44 | NR | NR | NR | NR | NR | NR |  |  |  |  |
| **Gao et al.** | Control | 35  (20/15) | 43. 6 土 5.0 | 26.5 ± 2.2 | 35.0 ± 4.6 | 35.3 ± 3.6 | 4.2± 0.5 | 126.0 ± 20.4 (ng/mL) | NR | NR | NR | NR |
|  | MAFLD | 96  (56/40) | 42.6 土 5.2 | 26.7 ±2.1 | 56.6 ± 11.0 | 53.5 ± 10.0 | 7.4 ±2.4 | 234.1 ± 56.4 (ng/mL) |  |  |  |  |
| **Hang et al.** | Control | 110  (62/48) | 44.75±6.32 | NR | NR | NR | 2.95 ± 0.36 | 28.38 ± 4.25  (pg/mL) | NR | NR | NR | NR |
|  | MAFLD | 200  (128/72) | 45.41 ± 6.9 | NR | NR | NR | 4.41 ± 0.68 | 43.46 ± 6.61  (pg/mL) |  |  |  |  |
| **Lai et al.** | Control | 29  (14/15) | 43.2 ± 14.2 | NR | NR | NR | NR | 12.9 ± 2.8 (ng/mL) | NR | NR | NR | NR |
|  | MAFLD | 60  (34/26) | 47.2 ± 11.8 | NR | NR | NR | NR | 24.9 ± 9.5 (ng/mL) |  |  |  |  |
| **Xing et al.** | Control | 22  (14/8) | 46.2 ± 7.3 | 22.1 ± 3.1 | NR | 30.3±6.8 | 1.6 ± 0.2 | 60.0 ± 2.4 (ng/mL) | NR | NR | NR | NR |
|  | MAFLD | 60  (40/20) | 48.3 ± 10.1 | 25.9 ± 1.9 | NR | 39.1 ± 7.8 | 2.5 ± 0.8 | 80.2 ± 7.6 (ng/mL) |  |  |  |  |
| **Sahar et al.** | Control | 15  (12/3) | 41.8 ± 7.1 | 23.9±2.2 | 26.1 ± 3.6 | 26.9 ± 6.0 | 0.7 ± 0.4 | 0.3 ± 0.2 (ng/mL) | NR | NR | NR | NR |
|  | NAFL | 15  (0/15) | 47.9 ± 4.3 | 34.6 ± 3.8 | 24. 3 ± 7.9 | 22.5 ± 8.0 | 12.1 ± 7.1 | 0.7 ± 0.5 (ng/mL) |  |  |  |  |
|  | NASH | 15  (5/10) | 50.2 ± 9.1 | 34.3 ± 5.0 | 73.7 ± 14.9 | 59.4 ± 9.4 | 12.7 ± 6.9 | 0.8 ± 0.5 (ng/mL) |  |  |  |  |
|  | MAFLD | 30  (5/25) | 49.1 ± 7.1 | 34.5 ± 4.3 | 49 ± 27.8 | 41.9 ±20.6 | 12.4 ± 6.9 | 0.8 ± 0.5 (ng/mL) |  |  |  |  |

ALT, alanine aminotransferase; AST, aspartate aminotransferase; BMI, body mass index; HOMA-IR, homoeostasis model assessment of insulin resistance; NAFL, non-alcoholic fatty liver; NAFLD, nonalcoholic fatty liver disease; NASH, non-alcoholic steatohepatitis; NR, not reported.

Data are presented as mean ± standard deviation or absolute frequencies. When the data were not presented as mean ± standard deviation in the original, they were properly transformed means and standard deviations by converting the median and quartile forms of chemerin levels [1].

**Supplementary Table 3.** Meta-regression analysis to assess the influence of continuous variables that between MAFLD and controls

| **Variables (NAFLD patients)** | **Adjusted *R^2^* (%)** | ***β*** | **95% CI** | ***P*** |
| --- | --- | --- | --- | --- |
| Sampe size | 7.4 | -0.005 | -0.125, 0.003 | 0.176 |
| Males/Females | 17.57 | 1.246 | -0.153, 2.646 | 0.077 |
| Mean age | 72.08 | -0.235 | -0.325, -0.145 | ＜0.001 |
| Mean BMI | -8.95 | -0.015 | -0.217, 0.186 | 0.867 |
| Mean AST levels | 25.5 | 0.069 | -0.003, 0.141 | 0.058 |
| Mean ALT levels | -2.84 | 0.020 | -0.034, 0.074 | 0.432 |
| Mean HOM-IR | -8.15 | -0.051 | -0.363, 0.261 | 0.726 |

ALT, alanine aminotransferase; AST, aspartate aminotransferase; BMI, body mass index; CI, confidence interval; MAFLD, metabolic-associated fatty liver disease.

**Supplementary table 4.** Risk of bias assessment using the Risk of Bias in Non-randomized Studies of Exposures

| **References**  **(Author, Year)** | **Confounding** | **Selection** | **Measurement of exposure** | **Departures from intended exposures** | **Missing data** | **Measurement of outcomes** | **reported result** | **Overall** |
| --- | --- | --- | --- | --- | --- | --- | --- | --- |
| Kukla et al.2010 | Moderate | Low | Low | Low | Low | Low | Low | Moderate |
| Sell et al.2010 | Serious | Low | Low | Low | Low | Low | Low | Serious |
| Yilmaz et al.2011 | Moderate | Low | Low | Low | Low | Low | Low | Moderate |
| Polyzos et al.2014 | Serious | Low | Low | Low | Low | Low | Low | Serious |
| Ye et al.2014 | Serious | Low | Low | Low | Low | Low | Low | Serious |
| Zhuang et al.2015 | Serious | Low | Low | Low | Low | Low | Low | Serious |
| Zwolak et al.2016 | Serious | Low | Low | Low | Low | Low | Low | Serious |
| Bekaert et al.2016 | Serious | Low | Low | Low | Low | Low | Low | Serious |
| Pohl et al.2016 | Serious | Low | Low | Low | Low | Low | Low | Serious |
| Lai et al.2017 | Moderate | Low | Moderate | Low | Low | Low | Low | Moderate |
| Kajor et al.2017 | Serious | Low | Low | Low | Low | Low | Low | Serious |
| Zhang et al.2018 | Serious | Low | Low | Low | Low | Low | Low | Serious |
| Hang et al.2019 | Moderate | Low | Moderate | Low | Low | Low | Low | Moderate |
| Sahar et al.2019 | Moderate | Low | Low | Low | Low | Low | Low | Moderate |
| Mohamed et al.2021 | Moderate | Low | Low | Low | Low | Low | Low | Moderate |
| Gao et al.2021 | Moderate | Low | Moderate | Low | Low | Low | Low | Moderate |
| Xing et al.2021 | Moderate | Low | Low | Low | Low | Low | Low | Moderate |

**Supplementary table 5.** Summary of the quality of evidence and strength of recommendation according to Grading of Recommendations Assessment, Development and Evaluation (GRADE) criteria

| **№ of studies** | **Design** | **Risk of bias** ^†^ | **Inconsistency** | **Indirectness** | **Imprecision** | **Other**  **considerations** | **Overall certainty of evidence** |
| --- | --- | --- | --- | --- | --- | --- | --- |
| NAFLD vs controls | | | | | | | |
| 15 studies | observational  studies | Serious | Very serious ^a^ | Not serious | Not serious | Serious ^c^ | ⨁◯◯◯  VERY LOW |
| NAFL vs controls | | | | | | | |
| 4 studies | observational  studies | Serious | Very serious ^a^ | Not serious | Serious ^b^ | Not applicable | ⨁◯◯◯  VERY LOW |
| NASH vs controls | | | | | | | |
| 4 studies | observational  studies | Serious | Very serious ^a^ | Not serious | Serious ^b^ | Not applicable | ⨁◯◯◯  VERY LOW |
| NASH vs NAFL | | | | | | | |
| 5 studies | observational  studies | Serious | Very serious ^a^ | Not serious | Serious ^b^ | Not applicable | ⨁◯◯◯  VERY LOW |
| moderate to severe steatosis vs mild steatosis | | | | | | | |
| 3 studies | observational  studies | Serious | Very serious ^a^ | Not serious | Serious ^b^ | Not applicable | ⨁◯◯◯  VERY LOW |
| present liver fibrosis vs absent liver fibrosis | | | | | | | |
| 4 studies | observational  studies | Serious | Very serious ^a^ | Not serious | Serious ^b^ | Not applicable | ⨁◯◯◯  VERY LOW |
| present lobular inflammation vs absent lobular inflammation | | | | | | | |
| 3 studies | observational  studies | Serious | Very serious ^a^ | Not serious | Serious ^b^ | Not applicable | ⨁◯◯◯  VERY LOW |
| present portal inflammation vs absent portal inflammation | | | | | | | |
| 3 studies | observational  studies | Serious ^a^ | Very serious ^b^ | Not serious | Serious ^c^ | Not applicable | ⨁◯◯◯  VERY LOW |

The level of importance for outcome measures

1. Serious inconsistency due to significant heterogeneity (*I^2^*＞50%)
2. Serious imprecision due to small sample size (n＜400)
3. Publication bias (if more than 10 studies) was detected by Egger’s test (P < 0.05) and funnel plot

**
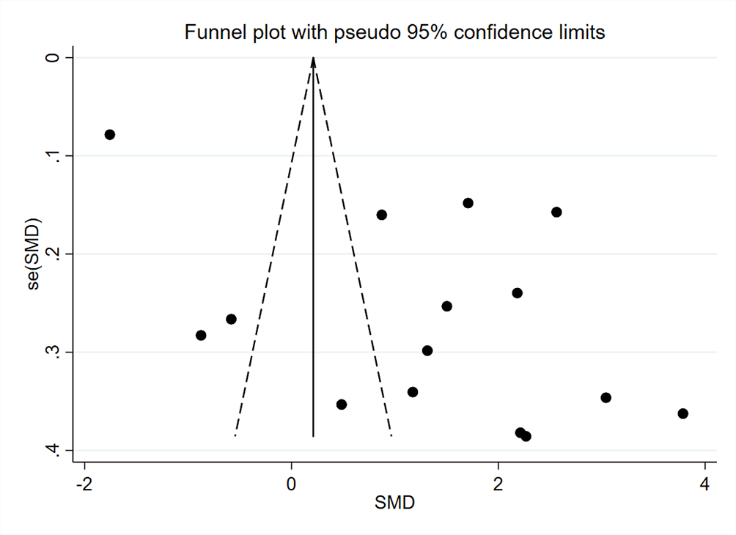
**

**Supplementary Figure 1** Funnel plots on the difference of circulating chemerin levels for the visual detection of publication bias between MAFLD and controls

## Reference

1. Hozo SP, Djulbegovic B, Hozo I: Estimating the mean and variance from the median, range, and the size of a sample. *BMC Med Res Methodol* 2005, **5:**13. doi: 10.1186/1471-2288-5-13.
